# TALK OF FAMILY

**DOI:** 10.7758/rsf.2024.10.5.07

**Published:** 2024-09

**Authors:** JESSICA HALLIDAY HARDIE, ALINA ARSENIEV-KOEHLER, JUDITH A. SELTZER, JACOB G. FOSTER

**Affiliations:** Hunter College and the Graduate Center, City University of New York, United States.; Purdue University, West Lafayette, Indiana and University of California, San Diego, United States.; University of California, Los Angeles, United States.; Indiana University, Bloomington, University of California, Los Angeles, and Sante Fe Institute, New Mexico, United States.

**Keywords:** family, social class, topic modeling, social institutions

## Abstract

We develop a novel application of machine learning and apply it to the interview transcripts from the American Voices Project (N = 1,396), using discourse atom topic modeling to explore social class variation in the centrality of family in adults’ lives. We take a two-phase approach, first analyzing transcripts at the person level and then at the line level. Our findings suggest that family, as represented by talk, is more central in the lives of those without a college degree than among the college educated. However, the degree of institutional overlap between family and other key institutions—health, work, religion, and criminal justice—does not vary by education. We interpret these findings in the context of debates about the deinstitutionalization of family in the contemporary United States. This demonstrates the value of a new method for analyzing qualitative interview data at scale. We address ways to expand the use of this method to shed light on educational disparities.

The family is a social institution that is central to most people’s lives (Rossi and Rossi 1990; Seltzer 2019; Stack 1974; Swartz 2009). Family is responsible for the care and rearing of the next generation. Throughout life, family members are important sources of socioemotional and economic support, both routinely and in times of crisis. Whether out of altruism, obligation, or necessity, family members are “there” for one another in many ways.

Social class, however, conditions the forms and roles of families in Americans’ lives, further increasing economic inequality. The deepest divide is between adults with and without a four-year college degree (Case and Deaton 2020). Adults with a college degree, for example, are more likely to marry and to stay married than those with less than a college degree (Smock and Schwartz 2020). They also have children later in life, on average, than those without a college degree (Guzzo and Hayford 2020). Also, given the still high degree of marital homogamy by education and the intergenerational transmission of resources, those with a college degree are likely to have more kin who similarly have college degrees and economic and social resources (Hirschl, Schwartz, and Boschetti 2023; Park, Wiemers, and Seltzer 2019).

Despite these structural differences, we do not know whether there are social class differences in how central family is in Americans’ everyday lives, or how family as an institution overlaps with other institutions that shape individuals’ lives. On the one hand, family may be more central in the lives of those who are not college educated. At least since the middle of the last century, ethnographic evidence suggests that working-class families have substantial social involvement with kin (Young and Willmott 1957; for a re-analysis of surviving data and a critique, see Lawrence 2016). Contemporary survey findings show that those who are economically disadvantaged spend more time with family members than those who are more advantaged (Bianchi and Vohs 2016). The greater proximity to kin contributes to the greater social involvement of the disadvantaged (Choi et al. 2020). The relatively weak social safety net in the United States also makes relying on family ties an essential way of getting by among the working class and poor (Seefeldt and Sandstrom 2015; Stokes and Patterson 2020; Swartz 2009). Thus greater centrality of family among those without a college degree may reflect the degree to which family members must rely on one another for support, and greater institutional overlap with family life among this group may reflect greater complexity in these arrangements.

On the other hand, changes in family structure and function may have contributed to fewer class differences in the centrality of family in American’s lives. Early theoretical debates about the overlap between family and other social institutions suggest that in Western industrial societies the family as an institution is separate from other social institutions, such as paid work and formal health care (Parsons 1943). This ideal type applied more to middle-class families than to working-class families in which wives and mothers were more likely to be employed and for whom family and other institutions had more overlap than middle-class families (Goode 1970). In the decades since these debates, both empirical evidence and a reconceptualization of the definitions of family (Furstenberg et al. 2020) have raised new questions about the extent of overlap between family and other social institutions and variation in overlap by social class. In particular, observations of the deinstitutionalization of marriage suggest a blurring of boundaries between family and the social institutions that organize other parts of social life (Cherlin 2004). These blurred boundaries and a context in which patterns of work are relatively more similar by gender now than in the past (Blau and Kahn 2017) may lead to similarities across social class in the extent to which family talk occurs in the context of talk about other institutions.

This article makes both substantive and methodological contributions. It shines new light on an important substantive concern: class differences in the centrality of family in adults’ lives. Evidence on the centrality of family in individuals’ lives typically comes from survey data or from in-depth interviews and ethnographies. The American Voices Project (AVP) combines survey and in-depth interview data for a large national probability sample (see Edin et al. 2024, this issue). Because the interviews are a larger corpus of qualitative data than would be available in other in-depth interview studies, the AVP provides a unique opportunity to apply a multistage, mixed-methods analysis of interview transcripts to describe class differences in individuals’ family experiences and how these are influenced by the major social institutions that structure individuals’ lives: health, work, religion, and criminal justice.

Methodologically, this article contributes by developing a novel application of a recently introduced topic modeling method, discourse atom topic modeling, or DATM (Arseniev-Koehler et al. 2022), to identify latent themes in the interview transcripts at scale. Topic modeling allows us to infer individuals’ maps of the social world from the rich qualitative interviews (Carley 1994). Our approach complements recent efforts to combine qualitative and machine-learning methods (Abramson et al. 2024, this issue; Bjerre-Nielson and Glavind 2022; Zilberstein et al. 2024, this issue). Our application is distinctive in combining word embedding methods with topic modeling to allow inductive identification and characterization of broad themes (topics) that can also be attributed reliably to individual segments of talk.

To adapt DATM for application to interview data, we adopted an iterative approach that combined DATM with traditional regression methods. Our interpretations of the DATM results and the regression analyses are enriched by an interpretive reading of full transcripts and samples of lines within transcripts. Our analysis proceeded in two phases. First, we used an inductive approach to the interview data, applying DATM to identify seventy-five topics emerging from the individual interviews. We interpreted three topics as family topics, which we combined into an overall family talk measure. We evaluated the face validity of the DATM topics based on a qualitative reading of transcripts from interviews. We then regressed the degree of family talk that interviewees engaged in during their interviews on their social class as indicated by their education and additional control variables. Finally, we selected cases based on regression residuals for a deep reading of transcripts. This first phase of our process revealed social class differences in the degree of family talk and found that family talk emerged in contexts marked by institutional overlap (family and work or family and health, for example), family complexity, and fictive kin language.

In the second phase of the analysis, we expanded our use of DATM to evaluate the existence and co-occurrence of additional latent topics. For theoretical reasons, we focused on how institutions overlapped with family talk at the line level rather than at the transcript level; DATM allows us to assess institutional overlap at this fine grain, where it is most likely to represent genuine blurring or overlap. To do this, we used machine learning to map the semantic space of the corpus again and to calculate the cosine similarity of each line in each transcript to 200 fine-grained topics. We then assessed the line-by-line association between institution talk and family talk in a multilevel regression and tested whether this association varied by social class. We confirmed that family talk was associated with the institutions of health, work, religion, and criminal justice (that is, where family was a topic of discussion, these other institutions often were as well) but found no evidence of moderation by social class.

Finally, we selected random samples of lines for deeper reading to assess how overlap between family talk and discussion of other institutions arose, as well as whether and how these discussions varied by social class. Our findings revealed that overlap often occurs because individuals seek to flexibly manage their engagement in other institutions to support their families. Whether through earned income, health insurance, social capital, or other strategies, individuals deliberately engage in three institutional domains (work, health, and religion) for family management or gain. Family can also bear the brunt of negative outcomes from these institutional domains, as well as in the criminal justice domain. Both engaging in other institutions for family support or managing the fallout of negative institutional engagement were common themes across social class, although those with a college degree typically had more resources and standing to secure better outcomes.

## The Centrality of Family in American Life

Although Americans are often depicted as culturally more oriented toward individualism than those in other countries (Fischer 2008), family is still a central organizing feature of their lives. Americans are born into and raised in families and maintain family connections throughout life. These family connections provide material support, emotional support, and social capital (Rossi and Rossi 1990; Swartz 2009). Yet how and to what extent family shows up in people’s lives varies by social class (Gerstel 2011; Hardie 2022; Lareau 2011). Those in the working class and those who are poor tend to describe spending more time with extended kin relative to the middle class and report greater practical assistance and emotional support exchanged between family members.

Research has attributed class differences in family support largely to differences in need (Gerstel 2011; Sarkisian and Gerstel 2012). Poor and working-class families often rely on private support to stay afloat (Kalil and Ryan 2010, Mattingly et al. 2021), although those who have economic resources are more likely to give financial transfers than those who are disadvantaged (Seltzer and Bianchi 2013; Swartz 2009). Private financial support, however, is vital for poor and working-class families because public safety nets such as Medicaid and Food Stamps can be cumbersome to navigate and a poor fit for families’ many needs (Danziger 2010; Paik 2021; Tach and Edin 2017). For middle-class and upper-middle-class families, family support helps maintain their class advantage through monetary gifts or loans, shared information, and connections to jobs (Collins 1998; Hamilton 2016). In some cases, simply knowing that family support is available if needed allows advantaged individuals to take more economic risks with bigger potential payoffs (Seltzer and Bianchi 2013).

Family life encompasses much more than the support family members give or need from each other. Family members spend time with each other in leisure activities, share meals that foster emotional closeness, and together develop ways of making sense of their world (Bianchi, Robinson, and Milkie 2006; Cheal 1988; DeVault 1994). Class differences in these dimensions of family life may vary in part because of class differences in the opportunities and constraints imposed by other social institutions, such as the organization of work or paid employment and of health care. What individuals say about their family lives and how much they talk about their families provide insight into the importance of family in their daily lives.

## Family and Other Institutions

An institution entails “a complex of positions, roles, norms and values lodged in particular types of social structures and organizing relatively stable patterns of human activity” (Turner 1997, 6). The family is an institution because it comprises multiple roles (spouse, mother, child, grandparent); norms (expectations of spousal romance, childrearing); and values (loyalty, heteronormativity) and because the family organizes patterns of human activity such as where and with whom individuals live and shared finances. The family, as an institution, also intersects with other institutions. Marriage is governed by the state and, for many, by religious institutions. Access to health care is organized in part by family ties and also by work, another institution that intersects with family by dint of time demands and financial remuneration.

Institutional overlap may not occur equally across social class, however. Prior work documents that poor, Black, and Hispanic families interact with the criminal justice system much more than middle-class, White, and Asian families (Johnson and Waldfogel 2002; Turney 2017; Turney and Wildeman 2013). Both work and unemployment structure family life differently by social class (Damaske 2011, 2021). Poor families also rely on some institutions to a greater degree for their survival, and this can lead to complex, multi-institutional involvement that can compound poverty (Paik 2021).

Although we know there are class differences in how families are enmeshed in other institutions, it is less clear whether connections between family and other social institutions affect the centrality of family in individuals’ lives. We explore these connections and family centrality by focusing on four institutional domains: health, work, religion, and criminal justice. Each of these was chosen based on deep readings of the transcripts in our first research phase, although they are also theoretically relevant and reflect prior research. Access to health care, as noted, is linked to families through public policy. Personal health status is also linked to others in one’s orbit and often managed by family members. Families manage leisure time around work, as we found in the transcripts, and sometimes family members work at the same locations. Religious institutions are organized around joint family involvement and often dictate normative aspects of family life (or try to). Finally, research has demonstrated that family members of those involved in the criminal justice system are deeply affected by that involvement themselves (Comfort et al. 2017; Turney 2017). Thus, these institutions are all theoretically enmeshed in family life. Our analyses identify the extent to which the institutions are combined with family talk.

## Research Questions

We pursue three questions: Do individuals who are disadvantaged talk more (or in different ways) about family than individuals who are advantaged? How are talk about health, work, religion, and criminal justice and individual characteristics associated with family talk in interviews? Do social institutions structure family experience more for those who are disadvantaged (no college) than those who are advantaged (college)? Together, our findings point to the ways that family and other institutions are linked in individuals’ lives and how these links may differ by social class.

## Data and Methods

We use qualitative and quantitative data from the American Voices Project, a national probability sample of adult householders, to understand the context of family talk during a given interview. The AVP is an omnibus survey combining a large number of holistic open-ended questions with closed-ended questions for a probability sample of adults in U.S. households between 2019 and 2021 (*N* = 2,349 completed interviews, of which 1,613 had been transcribed at the time of this project). The data include an oversample of households in high-poverty census tracts. Field work was in-person prior to the COVID-19 pandemic and then by telephone during the pandemic (American Voices Project 2021). The data are structured as interview transcripts and available under restricted conditions to protect individuals’ confidentiality (see Edin et al. 2024). We use a sample of 1,396 interviews, excluding those that were not in English or when the transcription procedures prevented us from distinguishing between interviewers and interviewees.

We analyze the data in two phases. In phase one, we explore the data at scale by adapting a recently developed approach to natural language processing (DATM) to the analysis of interview transcript data, with transcripts as the unit of analysis. DATM allows us to extract high-level topics from the corpus of transcripts and to construct an interview-level measure of family talk; we investigate its predictors at the interview level via multivariable regression and use those quantitative results to select cases for a deep qualitative dive into transcripts. In phase two, based on what we learn methodologically and substantively from phase one, we refine our approach to focus on fine-grained topics at the utterance (line) level. In that analysis, we treat these utterances (lines) as the unit of analysis but take into account the multilevel structure of the data in which lines are embedded in transcripts. Both phases of the analysis rely on DATM for construction of outcome variables and some predictors.

## Constructing the Outcome Variable: Family Talk

Phase one of the analysis estimates the amount of family talk based on the entire transcript of interviewee responses; in phase two, we use a finer-grained indicator of family talk by measuring it at the level of a specific utterance within a transcript. In other words, utterances (lines) are our units of analysis in phase two. To construct the outcome variables from the raw interview text, for both phases we use the discourse atom topic model. This approach allows us to construct measures of talk about other institutions (such as health or work) as well.

DATM has four steps. We outline them in depth because the method is novel; see Arseniev-Koehler et al. (2022) for the underlying theory and methodological details. The key idea is that DATM converts a document, such as an interview transcript, from a series of words into a series of fine-grained topics. Those topics are learned from a representation of the language in a corpus called a word embedding. Our DATM analyses use lines from interviewers and interviewees to maximize the input data. The statistical analyses of education differences in family talk use only lines from interviewees.

### Step 1: Constructing the Word Embedding

A word embedding assigns each word a position in a high dimensional space (sometimes called a semantic space); each word is therefore represented as a vector (a list of *D* numbers, where *D* is the dimensionality of the space). To construct a word embedding, we use a standard machine-learning algorithm called word2vec. Word2vec learns vector representations of words from the text data by performing one of two possible tasks: continuous-bag-of-words (CBOW) or skip gram (SG). For CBOW, the model predicts what a focal word will be, given a set of words around it, that is, its context. In practice, the model represents the context as the average of the corresponding word vectors; it identifies the word vector most similar to the average context (using cosine similarity) and predicts the corresponding word as focal. SG inverts this learning task, that is, predicting the context words from focal words.

Initially, word vectors are random and predictions are rarely correct. We train the model by converting the corpus into millions of these word prediction tasks; by gradually adjusting the word vectors to improve prediction performance, the model eventually learns a good embedding. In such an embedding, words that appear in similar company in the corpus tend to have similar word vectors.

The construction of word vectors involves several key choices. In both phases of analysis, we train word embeddings using a range of hyperparameters and then select the optimal model using standardized metrics for evaluating the quality of word embedding. Details on this step are in the appendix (see the section Training the Word Embedding).

### Step 2: Discovering Topics in the Embedding Space

To discover topics in the embedding space, DATM uses an approach called sparse coding (Arora et al. 2018). Sparse coding assumes that every word vector in the embedding can be written down as a sparse linear combination of a few fundamental or atomic units of meaning, that is, our topics. For example, the word *ring* might refer to a treasured family heirloom, the squared circle of professional wrestling, or the sound of a bell summoning ranch hands to dinner. We thus might expect the word vector for ring to be well approximated by an atomic unit having to do with jewelry, one having to do with sports, and one having to do with sound. Each of these atomic units, in turn, corresponds to a position in the embedding space: an atom vector. In both phases, to construct this decomposition of the word embedding, we use an algorithm called K-SVD (for K-Singular Value Decomposition), which generalizes the familiar K-means algorithm for clustering. The main hyperparameter for K-SVD is the number of atomic units, or building blocks K, that is, discourse atom vectors, which represent our topics in the embedding space. We select a final value for K (following Arseniev-Koehler et al. [2022]), seeking to balance performance across three metrics of topic quality (see the appendix section Topic Model Quality Metrics). In our phase one model, we prioritized coherent and diverse topics over coverage of the semantic space; we also favored a smaller number of total topics for interpretability, selecting a solution with K = 75 topics. In phase two, we emphasized coverage while retaining topic coherence and diversity; after testing values of K between 75 and 250, we selected a solution with K = 200 topics, which balances performance across the three metrics.

### Step 3: Interpreting and Labeling Topics

To interpret a particular atom vector, we look at the word vectors closest to it by cosine similarity. This approach is motivated by the generative model underlying DATM (Arora et al. 2016); the word vectors closest to an atom vector correspond to the words it is most likely to generate when viewed as a topic, that is, a probability distribution over words (for details, see the appendix section Interpreting Discourse Atom Vectors as Topics). In both phases, we identified the closest twenty-five words for each atom vector and their cosine similarities to the atom vector. On the basis of these most characteristic words, two of us labeled each topic with a low-level label indicating the specific content of the atom, such as psychological distress and, where applicable, a high-level label indicating broad topics like family, health, or work. We independently labeled the topics and resolved disagreement through discussion.

### Step 4: Converting Interview Transcripts to Topic Sequences

Our approach to converting the transcripts to topic sequences is motivated by the generative models underlying DATM (Arora, Liang, and Ma 2017; for a full discussion, see Arseniev-Koehler et al. [2022]).^1^ Inverting this generative model provides a recipe for taking each utterance and mapping it into a vector in the embedding space, that is, the region in the embedding space this utterance refers to, which Sanjeev Arora, Yingyu Liang, and Tengyu Ma (2017) call the local discourse vector. To compute this for each utterance, we first take the weighted average of the word vectors in each utterance; common words get lower weight and rare words get higher weight. Then we subtract the component that is common to all these utterance-vectors, which Arora, Liang, and Ma call the global discourse vector. The result now represents the utterance as a vector or position in the embedding space. This “utterance vector” can be compared with the word vectors and atom vectors in this space using cosine similarity (for details, see Arora, Liang, and Ma 2017; Arseniev-Koehler et al. 2022). Using this procedure, we take a given interview and compute a vector representation for every utterance.^2^

## Outcome Variables for Phase 1 and Phase 2 from DATM Steps

In phase one of the analysis, for all utterances in a given interview, we find the atom vector (topic) closest to the corresponding utterance vector. Thus we convert each transcript from a sequence of utterances into a sequence of topics.^3^ For example, an utterance reading, “I got married when I was twenty-two,” would be converted to a family topic. Once a given interview is represented as a topic sequence, it is straightforward to construct our initial percent family talk outcome variable. For a given transcript (now in the format of a topic sequence), we compute the percentage of topics in that sequence that have the high-level code family. This variable is measured at the person level and becomes the outcome variable in our phase one regression model (*N* = 1,396).

In phase two of the analysis, we adjust our approach to capture the possible co-occurrence of topics within responses, as noted. Specifically, we compute the cosine similarity of each utterance vector to all possible atom vectors (*N* = 200 atom vectors).^4^ This gives an estimate for how much each utterance in each transcript talks about each topic.^5^ Our outcome variable for phase two is each utterance’s average cosine similarity to the ten topics with the high-level code family. We identified these ten topics by two of us coding them as family related. The family outcome was the average cosine similarity across all ten topics to each line (for a list of topics averaged to create the family outcome, see table A.1).^6^ We labeled topics based on words with high cosine similarities and by reading the ten lines with the highest average cosine similarities for that topic. Here, our outcome variable and predictor variables based on topics are measured at the line level, using only interviewee lines (*N* = 375,161).

## Predictors of Family Talk

In both phases, we use the survey data and additional context variables to build regression models predicting family talk. We focus on describing the variables used in the second phase of the analysis because we present those results in more detail. We describe additional variables used in phase one in the appendix section Phase One Regression Model.

Our models include measures of socioeconomic status, demographic characteristics, and other contextual variables from the survey as well as five topic modeling-associated variables. From the survey, we include gender (woman = 1, man = 0), race and ethnicity (Black, Latino, White, and other), college education (four-year college degree = 1 or not = 0), age, and nativity (U.S. born = 1, not U.S. born = 0). We also include whether the interviewee is cohabiting or married, household size, and whether any children are living in the household to account for factors that might increase family talk mechanistically because interviewers asked about other residents in the household. We include a measure of urbanicity (categorized as living in a rural, suburban, or urban area) and whether the interview was conducted remotely by telephone (1) or not (0). Measures of socioeconomic status in addition to college education are: respondent employment status (1 = employed, 0 = not employed), home ownership (1) or not (0), logged annual household income (added to 1), and whether the respondent received any means-tested benefits^7^ or purchased health insurance through a marketplace (ACA) that provided subsidized premiums (1) or not (0).

We also include five measures assessed at the utterance (line) level. These include four measures of topics found in the transcripts, each of which is computed as the average cosine similarity of the line to each of a number of topics that all relate to the larger topic of health (12 topics), work (18 topics), religion (5 topics), and criminal justice (8 topics). For subtopics within each institutional category, see table A.1. In addition to these institutional measures, we include a measure of the number of words in each utterance.

## Analytic Strategy

In phase one, we used a variation of a mixed-methods approach, systematic anomalous case analysis (Pearce 2002), designed to use qualitative data to elaborate on regression model findings. The idea behind this approach is that researchers can first estimate a statistical model of a relationship using survey data, then run diagnostics to identify small and large residuals (differences between expected and actual values), and finally sample these cases and use qualitative data to explain why they do or do not adhere to the model. To do this, we first regressed the percentage of family talk in transcripts on demographic and household measures, family support and closeness, and socioeconomic status. These independent variables were based on theoretical concerns and a close reading of twenty-seven randomly selected transcripts.^8^ We then predicted family talk using the full model and produced residuals based on that model (r = actual family talk % minus predicted family talk %). Because our goal was to uncover potential explanations for the observed association between college education and family talk, we sampled forty-seven cases for which the models best predicted the outcome. We split these transcripts by gender and education (*N* = 17 transcripts among non-college-educated men, *N* = 14 among non-college-educated women, *N* = 16 for college-educated individuals).^9^ Two of us read and coded these transcripts, looking for instances of family talk within transcripts and the contexts in which they emerged.^10^ This qualitative analysis revealed three important conclusions regarding when family talk appeared in the context of interviews and motivated our analytic decisions in phase two.

We next turned our attention to a line-by-line analysis of transcripts to understand the differences in family talk by social class in more detail and by taking account of the co-occurrence of family and other institutions. With this new dataset—restructured transcripts to examine lines within individuals—we compared each family subtopic by social class to assess which subtopics were discussed more among those with a college degree and those without; combined family topics together using an average cosine similarity to each utterance and regressed them on the other institutional measures and individual characteristics; tested interactions between institutional predictors and college degree to assess potential moderation; and read subsamples of lines with medium and high levels of family talk and institutional talk.

All analyses in phase two accounted for oversampling and nonresponse by weighting descriptive statistics and the data in the multilevel models.

## RESULTS

The primary goals of these analyses are to examine class differences in family talk and its institutional context and to develop a new application of DATM to enhance analyses of large samples of qualitative interviews. The results we report address the substantive questions about family talk and illustrate the iterative approach required for the methodological innovation.

### Phase 1 Findings

Findings from the phase 1 regression showed that completing a college degree was significantly and negatively associated with how much individuals talked about family, net of the control variables. Our analysis of forty-seven sampled transcripts pointed to three contexts in which family talk emerged: family complexity and need, institutional overlap, and fictive kin language. We review these findings briefly.

#### Family Complexity and Need

Family talk often surrounded complex family relationships, such as the inclusion of stepparents, step-siblings, or step-children, expartners, and grandparents who played a parental role. To some extent, this family complexity may be mechanistically related to family talk, in that having more family members to discuss increases the time spent on those family-related discussions. Yet complexity in family relations also demanded attention and time to navigate. Interviewees related experiences managing relationships with step-kin, exes, and other relations for which there was no standard expectation of norms and obligations.

Family complexity was also intertwined with family need; in part, this is simply due to larger families having more members who need assistance. However, family instability is also linked to poverty, both as a precursor and consequence. These multiple and overlapping complex family relationships increased the prominence of family talk among those who were not college educated.

#### Institutional Overlap

Family members’ dual ties to institutions also appeared in the occurrence of family talk. Some respondents worked alongside their relatives, particularly spouses. Others volunteered or were otherwise involved in organizations in which family members worked or they lived in small towns in which interviewees shopped and ran errands in businesses in which family members worked. This institutional overlap was not limited to work. Health and access to health care were common themes in the interview transcripts, and they often overlapped with family talk when interviewees either helped family members who were injured or ill or were helped by them. We also found that discussions of religion and criminal justice involvement were related to family life. For religion, family members participated in religious organizations together. In addition, stories about criminal justice encounters often involved family members. This last finding was related to the interview structure, however, as the interviewers specifically asked participants about family members’ interaction with police. Overall, our findings from reading the transcripts did not suggest that institutional overlap was driving class differences in frequency of family talk, but that the contexts in which institutional overlap occurred may be associated with social class.

#### Fictive Kin Language

Finally, some interviewees described friends and community members in ways that evoke a kind of fictive kin relationship. Although in some cases these were close family friends that were considered to be like family, in many cases they were not. For example, respondents sometimes reported on work colleagues being “like family.” Some respondents also used family-related language in a negative manner or while simultaneously distancing themselves from the people they were describing. Thus, family language was used descriptively to compare or characterize other kinds of relationships.

### Phase Two Findings

We designed the second phase of the analysis to more deeply examine class differences in family talk, how talk about other institutions overlapped with family talk, whether social class moderated the degree of overlap between family talk and talk about other institutions, and the context in which this overlap occurred. We focused on the theme of institutional overlap both because of its theoretical importance to the study of family and because the AVP study lent itself to this focus more readily than the other two emergent themes (family complexity and fictive kin language).

#### Descriptive Statistics

The first part of table 1 presents descriptive statistics for the variables used in our analyses at the individual level and at the line level. The final six measures are characteristics only of transcript lines. Once weighted, the sample is well balanced by gender. More than half self-identified as White, just over 10 percent as Black, and almost 17 percent as Hispanic or Latino. Nearly one-third had completed college. Respondents were between the ages of eighteen and ninety-four, averaging 47.6. A large majority (88.1 percent) were born in the United States and slightly more than half were living with a romantic partner. More than half were employed and additional analysis (not shown) suggested that the largest groups of unemployed persons were retired and disabled. About 29 percent of households included children under the age of eighteen; the average household size was 2.4 persons. Slightly fewer than half of the interviews were conducted remotely and 48.5 percent of those interviewed owned their own home. Average (nonlogged) household income was more than $51,000 per year. About 35 percent received at least one form of public support. Characteristics of individuals and of lines are very similar.

#### Family Subtopics by Social Class

Table 2 presents the mean values of each family subtopic by social class. At the line level, the zero-order association between the family talk subtopics and social class is minimal. Four subtopics did not differ at all by whether participants had a college degree (younger generation, formal transitions, family history, and children). Zero-order levels of family talk differed slightly by social class for other topics. College graduates spoke more about holidays, tracing lineage, and transition moments than those without a college degree. Those without a college degree spoke more about kinship, abuse and conflict, and older generations than those with a college degree.

#### Family Talk and Institutions

Our topic modeling approach also revealed a great deal of institution-related talk, allowing us to test the proposition that institutional overlap drove family talk and that this might explain differences in family talk by social class. Table 3 presents the findings from the hierarchical linear regression analysis predicting family talk by line based on individual characteristics and the presence of other institutional talk within each line. Our modeling strategy accounts for how lines are grouped within individuals by including a random intercept at the individual level. In preliminary analyses, we tested the inclusion of a random coefficient for college degree but it was not warranted based on a likelihood ratio test. Overall, variability by line was considerably higher within individuals than across individuals, as indicated by the intraclass correlations. However, individual characteristics, including social class, still had statistically significant net associations with family talk at the line level, suggesting that some class differences were suppressed in the zero-order associations in table 2.

As table 3 makes clear, we found that social class and institutional talk were both significantly associated with family talk in models 1 and 2. When including both in model 3, the magnitude of the coefficient for a college degree declined slightly but remained negative and statistically significant. In contrast, the coefficients for institutional talk remained virtually the same. Institutional talk was significantly and positively associated with family talk. For example, in model 3, a one-unit increase in the average cosine similarity of health topics predicted an increase in the average cosine similarity of the family topic by 0.098. A one-unit increase in the average cosine similarity of work topics, religion topics, and criminal justice topics also predicted net increases of 0.059, 0.040, and 0.418 in family talk, respectively. We suspect that the criminal justice coefficient was larger than the coefficients for other institutions because of the way the interview was structured; respondents were specifically asked about their family members’ experiences of arrest.

We next investigated whether the associations between other institutions and family talk were stronger for non-college-educated interviewees than for their college-educated counterparts. To do this, we interacted college with each institution variable (health, work, religion, and criminal justice). None of the interaction terms was statistically significant, suggesting that although social class and institutional talk were both independently associated with family talk, the degree to which the institution variables were associated with family talk did not vary by social class.

As a last step, we examined transcript lines to understand the contexts in which talk about family overlapped with talk about health, work, religion, and criminal justice. We also remained attentive to possible class differences in the content of these discussions. To do this, we randomly sampled twenty lines each at the low (<50th percentile), medium (between the 50th and 75th percentile), and high (>75th percentile) levels of family talk and each of the institutional variables, separately by college degree or no college degree. This means for each institution of health, work, religion, and criminal justice, we sample 360 lines (180 for non-college-educated interviewees and 180 for college-educated). We then read these lines for themes and meaning, seeking to understand how family talk arose within the context of other institutions and whether these contexts differed by social class. We examined both the sampled lines themselves and also returned to the transcripts to identify their broader contexts. Based on a preliminary reading of lines, we focused on those with medium or high average cosine similarity to our topics of interest. Those with low cosine similarity to family, for example, could be about any other topic and did not reveal any meaningful patterns within the data.

#### Family and Health

Utterances with above-median cosine similarities to both family and health topics typically touched on both topics very clearly. We noted three themes in the ways these topics arose within interviews: fallout from family members’ medical conditions, substance abuse, and managing well-being among couples. These themes arose among both non-college-educated and college-educated interviewees.

Fallout from health problems often occurred in the context of multiple institutions. Patrice, a college-educated Black woman, for example, described how her family’s health problem led to her decision to stop working. Further, when she was younger, she helped her grandparent take care of a relative with a serious illness. Patrice’s career and educational trajectories were delayed by these and other family-related tragic events, but eventually she completed a graduate degree and had a thriving career. Anthony, a middle-aged Hispanic man also described his life being changed dramatically when his wife died in a car accident. As he described it, it was the most painful experience he ever had, and he went through therapy and years of mourning.

Another strong theme was substance use, which also sometimes intersected with other institutions. For example, Nicole, a low-income, non-college-educated young Hispanic woman, explained that her mother had legal problems because she refused to testify against someone who was accused of a drug crime. In other cases, participants described family members who overcame drug addiction. A non-college-educated young White man, for example, described how his sister’s pregnancy led her to abstain from drugs: “I guess just getting pregnant kind of woke her up to reality somehow and she by some miracle was able to get clean.” These discussions were not limited to those without a four-year college degree. Ellen, a young White woman with a college degree, for example, described that she “struggl[ed] on and off for years with addiction issues.” The repercussions of such drug and alcohol use seemed less severe among the college-educated than the non-college-educated group, but this may be in part a difference between those who were able to complete their educations in addition to becoming drug-free relative to those who were not.

Finally, some interviewees discussed managing well-being along with their romantic partners. As Rosa, an elderly Hispanic woman without a college degree who had recently celebrated her fiftieth anniversary with her husband, remarked, “We [she and her husband] have our health. We are well, thank God.” Nicole, a middle-aged Hispanic woman without a college degree, also said that now that her husband was over a recent illness “we’re seeing the doctor regularly, so we’re good right now.” Other interviewees mentioned looking forward to years with their significant other, with the implicit expectation that both would be healthy.

#### Family and Work

We anticipated a clear association between work and family talk in the more nuanced analysis of text in the second phase. Although utterances with high cosine similarity on family and work are proximate in semantic space, the text did not always include both topics explicitly. There are, however, clear examples in which both work and family are discussed. Work was sometimes discussed as a means to support family. Ashton, a young, college-educated White man, for example, described saving money from his earnings “because one day I plan on buying a house and a car and supporting a family.” Similarly, Anna, a young college-educated White woman, described staying “on top of the payments” and avoiding debt as her responsibility within her household.

Relatedly, many interviewees, both college-educated and non-college-educated, spoke about work and family in relation to money. Anh, for example, was an elderly immigrant who was not college educated but had managed, with her husband, to establish a middle-class life in the United States. She described how she had handled the household’s finances by herself and that her husband “didn’t know about money. . . . He was very surprised when I said I bought [a] house.” When the couple was employed in their home country, Anh made more money than her husband, but in the United States he had worked for a large, profitable company and they had bought a house and raised children who were all thriving.

Finally, work was sometimes discussed in the context of giving to or receiving help from family members. Albert, a college-educated Hispanic man in his early sixties, told the interviewers that he and his wife worked hard to accrue savings to take their daughter on an international trip for her birthday. Troy, a non-college-educated Black man in his sixties described how his family “came up kind of hard. . . . So, we just came up, had to work, weren’t no play times, we had to help my parents.” In a very different context, Liana, a young White college-educated woman, described how her parents had “switched insurance providers to make it easier for me to see my doctors without us petitioning as much.” Liana had a chronic health condition that required frequent doctor visits and multiple medications, making health insurance a critical resource, and she was able to manage this resource through her parents’ work.

#### Family and Religion

Religion and family are perhaps an unsurprising overlap. Religious institution membership is rarely individual; families typically join a religious congregation as a unit and parents typically organize their children’s religious (or nonreligious) upbringing. Suzanne, an elderly non-college-educated White woman, reported that her grandchildren did not go to Catholic school like she had: “But those kids didn’t go to Catholic school. And I just figure it’s their choice.” Tabitha, however, a college-educated Hispanic woman in her late thirties, was deeply involved in her faith, going together with her husband and son to Bible study. Her daughter attended the church’s youth group, and Tabitha reported, “She likes it though, because she was like, ‘Are we going to church today?’” These decisions about religious service membership and involvement were family affairs.

Religion also played a practical role in individuals’ lives, either as a source of solace or as a way to garner resources through access to a community. Carolyn, a White woman in her sixties without a college degree, explained that when her husband died, her children and grandchildren were very upset so she made a memorial to him: “We’re getting over it. So that’s why I keep his picture up there, and they say good morning to him. They tell him good night, and they pray.” Tom, a White college-educated man, and his wife used church connections to adopt their children.

#### Family and Criminal Justice

Discussions around criminal justice were often about more than interactions with formal institutions. Family and criminal justice sometimes co-occurred in descriptions of family members as victims of crimes. Felix, a young non-college-educated Hispanic man, for example, talked about how he had felt shame when a camera given to him by his relatives was stolen from his car. To hide what happened before a family trip, he and his wife bought a new camera, despite the expense. Carl, a middle-aged Black man who had not completed college, described how his father was murdered when Carl was young: “Do you believe that? . . . But it, whatever man. . . . He got murdered.” This was not Carl’s, or his father’s, first experience with crime. His father had been in and out of jail throughout his young life.

Some interviewees spoke about police encounters, often when prompted by interview questions. Alex, for example, was a middle-aged White man in a professional occupation. He described being embarrassed once when he was stopped for speeding and then taken in a police cruiser to a neighborhood where he was known. The next time he was caught speeding (as he told the interviewer, this was because he was late to work), Alex decided he would use his work position to avoid a ticket or more embarrassment, explaining that he said to himself, “‘I got to try this [emphasize his work position to the police officer].’ It actually worked.” As he explained to the interviewer, he noticed that the police like (or perhaps respect) the uniform he was wearing.

## DISCUSSION

This article explores the extent, patterning, and context of family talk by social class with a novel, nationally representative dataset of in-depth interview transcripts as both a corpus for a new application of a recently developed machine-learning approach and a set of meaning-making accounts. We make two important scholarly contributions with this work. First, our findings reveal important social class differences in the extent of family talk in transcripts overall and that family talk often occurs in the context of family complexity and need, institutional overlap, and fictive kin language (Lareau 2011; Paik 2021). Furthermore, we do not find evidence that institutional overlap occurs differently by social class, lending some evidence to theories of deinstitutionalization of family life. Second, our article represents a significant methodological advance in the application of machine-learning methods to interview data. Because DATM can learn focused, fine-grained, high-quality topics from a small corpus with specialized vocabulary—and connect these topics to small semantic units like interview responses—it is a promising tool for mixed-method research using interviews, combining the power of popular word embedding methods with the synoptic scope of topic modeling. The article demonstrates the extension of these methods to interviews and shows the detailed insight it provides at the utterance level.

A deep reading of transcripts in our preliminary analyses revealed that family talk occurs in the context of complex family relationships and need, institutional overlap, and the language of fictive kinship. Conversely, we may think of low levels of family talk as growing out of the ability to operate as an individual in family life—family relationships are more streamlined and require less attention, encounters with many institutions occur mostly on one’s own, and family as a reference to a type of relationship is less salient. These findings point to the mechanisms by which family is central to some Americans’ lives more than others. They also suggest a fruitful intersection between the analysis of individual agency (Zilberstein et al. 2024) and the prevalence of family talk.

Our next set of findings built on what we learned from phase one about differences between transcripts. By focusing on line-level data in this second phase, and mapping a more detailed picture of the semantic space, that is, one with more topics, these analyses offered us more analytic nuance. First, we confirmed net class-based differences in family talk overall, where family was more often a topic of lines for those with less than a college degree than those who had a college degree. Second, we tested the association of four institution-related topics (health, work, religion, and criminal justice) with family talk using hierarchical linear models to evaluate more rigorously our preliminary findings. We found confirmation that institutions are overlapping in individuals’ lives, with talk of these four institutions and family closely associated with one another at the line level. We found no evidence, however, that the strength of the links between family talk and talk about other institutions varied by education. The similarity in institutional overlap between those with and without college degrees is consistent with the notion that family ties have become deinstitutionalized (Robbins, Dechter, and Kornrich 2022).

A close reading of transcript lines points to three conclusions. First, individuals manage their engagement in other institutions to support family life. This is notably true for work, where earnings and benefits can be employed to help family members, but also for religious institutions, which are sources of social capital, and health, where good health is seen as a resource or asset in families. Second, families manage the fallout resulting from disruptions from other institutions. This was particularly (but not exclusively) shown in health problems and encounters with criminal justice, where family members helped one another in times of hardship or mourned their losses. Third, although we did not systematically observe social class patterns, some individual stories illustrated the way privileges, such as signals about having a high-status job, allowed college-educated individuals to escape difficult situations or to manage fallout. Overall, we see how family members are a source of support for each other, helping each other with health problems and other needs. The persistent importance of family support suggests that some aspects of family life remain institutionalized (Cherlin 2020).

Our work also offers insight into the application of machine-learning methods, especially topic modeling, to interview transcript data. Other contributions to this issue (Abramson et al. 2024; Zilberstein et al. 2024) used similar machine learning methods—that is, word embeddings—as part of their analytic strategy. However, these papers used embeddings either to broaden the language used to operationalize a focal concept such as pain (Abramson et al. 2024) or to represent a concept such as agency (Zilberstein et al. 2024) and to measure similarity of interviews or segments to that concept. We use embeddings as part of a powerful new approach to topic modeling, allowing us to proceed more inductively and to discover a range of nuanced articulations of key institutions in the interviews. Discourse atom topic modeling allows us to represent topics and interview segments in a common semantic space and quantify the similarity of topics and segments. Although this is similar (at a high-level) to the concept mover’s distance approach of Zilberstein and colleagues, DATM emerges from the theoretical machine-learning literature (Arora et al., 2016, 2018; Arora, Liang, and Ma 2017; Arseniev-Koehler et al. 2022) and is close to standard word embedding approaches, making the full machinery developed over the past decade applicable, such as methods for assessing robustness, as in Arseniev-Koehler and Foster (2022). Our topic modeling approach enabled fine-grained analysis of themes in the interview transcripts, even though the large size of the corpus inhibits thematic qualitative analysis of the full dataset using traditional methods. We emphasize, however, that although topic modeling guided our analyses, focused qualitative analyses were still crucial to unpacking nuances in our quantitative results; we share this close collaboration between computational and qualitative analysis with both Corey Abramson and colleagues (2024) and Shira Zilberstein and colleagues (2024).

To better answer our research questions, we also found we needed to revise how DATM assigns topics to the utterance data to explicitly consider co-occurrences between topics within utterances, an innovation in the second phase of our analysis. Our approach is more efficient than earlier approaches that assign single topics to a strip of text and then use sliding windows to account for potential co-appearance of topics within a larger unit (Arseniev-Koehler et al. 2022).

Although our results clearly indicate the promise of DATM as a method for analyzing interview data at scale, our experience working with the AVP data also suggested some ways that qualitative data like interviews could be prepared to make such analyses easier and more reliable. Standardized transcription practices would make such data more amenable to natural language-processing methods. These include uniform conventions for labeling interviewer, interviewee, and (where needed) translator and systematic approaches to masking personally identifying information (direct and indirect) that nonetheless allows stable reference. For example, when multiple personal names are used in a response, they should be differentiated and systematized across lines, ideally in ways that indicate familial relationships like the parent or child of the interviewee. Such up-front investments will make it vastly easier to apply machine-learning methods reliably while preserving confidentiality. This is an especially pressing need given the new National Institutes of Health policy on sharing qualitative data (DuBois et al. 2023), which will increase the availability of qualitative data and likely increase the application of computational methods to such data.

Our study has limitations. The AVP interview asks explicit questions about family and institutions, which may raise concerns that this drives our findings. More broadly, a challenge of applying topic modeling to interviewee data is how to account for the role that interview guides and interviewers play in the topics brought up by interviewees. Among our results, most vulnerable to this criticism are connections between family and occupational inheritance (an aspect of work) and between family and experiences with police (an aspect of criminal justice). However, most of the interview questions do not ask explicitly about the connections between family and institutions. Another limitation is that the AVP does not ask about most family members who live apart from the interviewee, and some questions ask about family and friends combined without distinguishing between the two. The AVP open-ended responses enrich the survey content on these family-related topics, and, with the DATM method, allow a deeper dive into the role these kin may play in individuals’ lives even without direct interview questions.

Finally, family talk is, of course, a generalized indicator. Family talk may be both positive and negative. Particularly among families where need is high, family talk included both closeness and conflict. Thus we do not assume that family talk is synonymous with family closeness; findings from our phase one regression and qualitative analyses support this. Instead, family talk indicates a more or less intense presence of family as an institution and reference point in people’s daily lives. In addition, family may be central even when people do not talk about it very much. To some extent, this measure represents the degree to which family is central as an acknowledged presence in individuals’ lives. For instance, the college educated may not discuss their family very much but the resources they receive from kin and the safety net the family provides, even if it is not drawn on, are a major way families transmit advantages such as college degrees (Swartz 2009).

Our approach focuses on family and institutional connections as if they are in a steady state. Yet the AVP data were collected, in part, during the COVID-19 pandemic. Family and institutional responses to this exogenous shock may affect the degree to which family members’ institutional ties and the content of those ties vary by class. Although we control statistically for whether the interviews occurred after the start of the pandemic by using an indicator variable for whether they were conducted remotely, a fruitful line of inquiry would be to examine whether the pandemic altered talk about family and other institutions. As Max Besbris and his colleagues (2024) find, the pandemic—a health-related event—created housing shocks that some could alleviate through family support while others could not. Work by Catherine Thomas and her colleagues (2024) finds similar social class differences in the impact of the pandemic, particularly in the areas of home (family) life, work, and health.

Our approach and findings contribute to the literature on family and inequality using unique data and an innovative expansion of discourse atom topic modeling to examine how individuals’ accounts reveal family involvement and how this involvement is patterned by social structure. Our findings point most prominently to the value of a multi-institutional approach to studying individuals’ family lives and the factors that create and perpetuate disadvantage. They also offer clear methodological templates for further application of DATM to the type of rich interview data that projects such as the American Voices Project provide.

## Figures and Tables

**Table 1:** Weighted Descriptive Statistics

	Individual-Level Mean/% (*N* = 1,396)	SE	Line-Level Mean/% (*N* = 375,161)	SE

Woman	49.6%		49.5%	
**Race and ethnicity**				
Black	10.3%		9.6%	
Hispanic or Latino	16.7%		16.1%	
White	63.5%		64.2%	
Other	9.5%		10.2%	
College	31.1%		30.4%	
Age	47.6	0.95	48.6	0.91
US born	88.1%		88.5%	
Coupled	52.7%		54.8%	
Household size	2.4	0.07	2.5	0.09
Children <18 in household	29.4%		29.29%	
**Urbanicity**				
Rural	21.4%		23.8%	
Suburban	51.2%		51.2%	
Urban	27.0%		25.0%	
Interview remote	45.3%		41.8%	
Employed	56.3%		56.3%	
Income (logged)	7.6	0.24	7.6	0.28
Home owner	48.5%		50.1%	
Public support	35.4%		37.6%	
**Characteristics of lines**				
Family talk	-	-	0.27	0.95e-3
Health talk	-	-	0.26	0.83e-3
Work talk	-	-	0.24	1.02e-3
Religion talk	-	-	0.25	1.07e-3
Criminal justice talk	-	-	0.30	1.17e-3
# of words in an utterance	-	-	45.31	1.15

*Source:* Authors' calculations.

*Note:* Variables defined in the text. Percentages may not sum to 100 percent due to rounding.

**Table 2:** Cosine Similarity to Lines for Each Family Subtopic, by College Degree (*N* = 375,161)

Subtopic Label	No College	College	Top Twenty-Five Words

Holidays	0.20	0.21	Thanksgiving, christmas, easter, holiday, wedding, Halloween, weddings, dinners, celebrate, celebration, reunion, birthdays, celebrated, anniversary, birthday, 50th, cookout, get-togethers, presents, weekend, barbecue, cookouts, christmases, barbecues, celebrating
Kinship	0.33	0.32	Son, daughter, nephew, wife, grandson, husband, granddaughter, niece, girlfriend, sister, boyfriend, fiancé, brother, cousin, stepdaughter, sister-in-law, stepson, brother-in-law, daughters, sons, oldest, ex-husband, daughter-in-law, youngest, fiancée
Younger generation	0.31	0.31	Nephews, grandchildren, grandkids, kids, sisters, nieces, girls, boys, brothers, moms, siblings, sons, granddaughters, cousins, great-grandchildren, babies, aunts, dads, daughters, grandbabies, uncles, stepchildren, grandsons, wives, daddies
Formal transitions	0.33	0.33	Baptized, adopted, divorced, faithful, supported, happily, married, fought, committed, sexually, forgave, remarried, marriage, separated, divorce, virgin, raped, engaged, parental, acknowledged, welcomed, reconcile, wedlock, molested, marrying
Family history	0.23	0.23	Paternal, great-grandmother, grandfathers, great-grandfather, maternal, half-sister, aunties, step-sister, adored, grandmothers, dysfunctional, half-brother, dutch, disowned, pancreatic, adoptive, piedras, elder, grandmas, cajun, cerebral, Zacatecas, deceased, estranged, Brazilian
Abuse and conflict	0.18	0.17	Abusive, manipulative, jealous, violent, arguments, narcissistic, temper, sexually, disagreement, angry, volatile, cheated, confrontation, argument, outspoken, aggressive, fight, narcissist, unstable, aggravated, anger, verbally, arguing, altercation resentful
Tracing lineage	0.20	0.21	In-law, deceased, backbone, ours, great-grandmother, belongs, in-laws, grandparent, inherited, manages, remains, maintained, elder, inherit, supportive, paternal, stepchildren, separate, attached, aunts, theirs, grandfathers, father-in-law, dies, stepbrother
Children	0.24	0.24	One-year-old, four-year-old, 14-year-old, 15-year-old, six-year-old, two-year-old, three-year-old, seven-year-old, nine-year-old, girly, 12-year-old, 10-year-old, 19-year-old, eight-year-old, firstborn, toddler, 11-year-old, 13-year-old, oldest, baby, boy, spoiled, boys, littlest
Transition moments	0.32	0.33	Divorcing, settled, finalized, '85, separating, newly, eventually, settling, '84, '86, redoing, shortly, 2010, '98, 1992, moved, '93, finishing, '91, '94, '81, '82, remarried, '89, meanwhile
Older generation	0.38	0.37	Mother, grandmother, grandma, mom, dad, father, aunt, grandfather, grandpa, stepmom, stepmother, stepdad, grandparents, sister, daddy, stepfather, mum, godmother, mother-in-law, mama, uncle, parents, grandmothers, stepdad, paternal

*Source:* Authors' tabulation.

*Note:* Lines analyzed from 1,396 transcripts.

**Table 3:** Weighted Hierarchical Linear Models Predicting Average Line Cosine Similarity with Family Topic (*N* = 375,161)

	Model 1	Model 2	Model 3

Woman		0.004*** (0.001)	0.007*** (0.001)
**Race**			
Black		−0.006** (0.002)	−0.002 (0.002)
Hispanic or Latino		0.000 (0.002)	0.003 (0.001)
Other		−0.001 0.002	−0.001 (0.002)
College		−0.004** (0.001)	−0.003** (0.001)
Health talk	0.098*** (0.006)		0.098*** (0.006)
Work talk	0.058*** (0.008)		0.059*** (0.008)
Religion talk	0.040*** (0.004)		0.040*** (0.004)
Criminal justice talk	0.418*** (0.007)		0.418*** (0.007)
Constant	0.089*** (0.002)	0.245*** (0.004)	0.086*** (0.004)
σ^2^b	0.000	0.000	0.000
σ^2^w	0.003	0.004	0.003
ICC	0.046	0.034	0.027

*Source:* Authors' calculations.

*Note:* Standard errors in parentheses. Models 2 and 3 also control for age, U.S. born, cohabiting or married, household size, urbanicity, children in household, interview conducted remotely, employment, logged income, home owner, receiving public support, and number of words in utterance. Model 1 also controls for number of words in utterance. Lines analyzed from 1,396 interviews.

* *p* < .01;

** *p* < .001;

*** *p* < .0001

## Footnotes

1 This model assumes that words in a sentence or utterance are selected based on both their raw frequency and on what is currently being talked about in the text, that is, the discourse vector; further, the discourse vector is a combination of a fixed global discourse vector and a variable local discourse vector.

2 We also exclude utterances with fewer than five tokens because estimating the underlying topic is often very noisy and ambiguous for very short utterances.

3 In essence, this representation treats each utterance as produced by a single topic—the topic that is the closest approximation of its utterance vector.

4 We do this rather than merely finding the atom vector closest to the corresponding utterance vector, as we did in phase one.

5 More technically, this calculates the projection of the utterance onto each atom vector. Natural language processing (NLP) researchers have long quantified the “connotations” of a particular word by projecting it onto other words or onto latent dimensions (like gender). Alina Arseniev-Koehler and colleagues (2022) extended this approach to topics, measuring and validating the gender connotations of various topics. If we imagine an utterance that talks almost exclusively about a single topic (say, family members), its utterance vector will be very close to the corresponding atom vector and more distant from others. If, by contrast, an utterance combines talk about family and finances, its utterance vector is likely to be somewhat close to the atom vectors for (some) topics in both high-level categories, and more distant to others. We can heuristically think of this as the amount of talk on a particular topic in the utterance; more literally, we can view it as capturing how much the utterance connotes each topic.

6 We computed Cronbach’s alpha for each broad topic and tested alternatives to average cosine similarities in the model-building phase. Cronbach’s alpha for our broad family measure was 0.92. For the other institutions it was similarly high: health = 0.89, work = 0.91, religion = 0.92, and criminal justice = 0.91. Alternative measures did not alter our findings.

7 Programs included: TANF, SNAP, housing vouchers, SSI, WIC, subsidized childcare, free or reduced-price lunch, Medicaid, CHIP, and public housing.

8 We initially sampled twenty cases, one of which was disqualified due to having more than one interviewee, stratified by gender and education and limited in age to those ages forty-five to sixty-four. We limited the age range to a group we expected would have the most obligations to and interactions with family. We added another eight cases later at the low and high ends of education to further confirm our initial impressions.

9 We first identified case IDs with residuals closest to 0 and selected the forty closest values. We then had to oversample low absolute value residual cases for college-educated men because we only had two transcripts that fit into this group at the first pass.

10 We also read transcripts for a small number of cases with large residuals, the content of which showed little discussion of family issues.

## APPENDIX

The following sections offer additional details on how we conducted our DATM and regression analyses.

### Training the Word Embedding

Before training the word embedding on the transcript data, we cleaned the data by tokenizing it and lowercasing all characters. We decided to retain selected punctuation in both phases of the analysis, after observing that it was used in informative and systematic ways; for example, we observed that ellipses appeared to indicate pauses or hesitation in the transcript. We also decided not to remove digits which we observed to be informative (for example, indicating ages, parts of abbreviations, or sums of money). Each utterance (line) in the transcript was considered a sentence to input into Word2Vec. For training the word embedding, we included lines from interviewers and interviewees; this is because, in general, more data lead to higher quality embeddings.

The main hyperparameters of the embedding are the training task (CBOW or SG), dimensionality of the semantic space *D*, and the size of the context window *n* (the number of words to either side of a target word that goes into its context). We trained embeddings with a standard, fixed dimensionality *D* = 200, for each of the four possible combinations of context window (*n* = 5, 10) and training task (CBOW or SG).^11^ We then tested whether our model improved with reduced dimensionality (*D =* 100). Following standard practice, we chose our final model based on a standard metric of embedding quality: performance on WordSim-353 (which compares the model’s judgments about word similarity to human raters). In phase one, the final word embedding model was trained using *D* = 200 and in phase 2, the model was trained using *D* = 100. In both phases 1 and 2, the final models were trained using the SG learning task,^12^ and *n* = 10, and had Spearman correlations of 0.56 (*p* < .0001) and 0.56 (*p* < .0001) to human ratings, respectively. This is considered strong performance on such evaluations. In both phases, we also only learned word vectors for words occurring at least twenty-five times in the corpus to prevent learning low-quality word vectors, and set the number of epochs (that is, training iterations over the corpus) for the training algorithm at ten.

### Topic Model Quality Metrics

Following Arseniev-Koehler et al. (2022), we use three topic model quality metrics to select the number of topics: topic coherence, topic diversity, and topic coverage. Topic coherence measures whether the most probable words for a particular topic are close to one another (in cosine similarity). Topic diversity measures the text to which the most characteristic (probable) words for each topic are distinct and not overlapping. Coverage evaluates how well the sparse coding explains the full embedding space, using *R*^2^ as a metric. In the first phase, after testing values of K between 7 and 800, we found that a solution with K = 75 topics balances coherent, diverse topics with good coverage. After working with the topics in the first phase, we decided to place higher relative value on coverage (which, in turn suggests a higher number of topics). Thus, in the second phase, after testing values of K between 75 and 250, we found that a solution with K = 200 topics prioritizes coverage while still providing coherent and diverse topics.

### Interpreting Discourse Atom Vectors as Topics

Drawing on the theoretical machine-learning literature, we can use a simple generative model to characterize each discourse atom vector as a topic. The latent variable model of Arora et al. (2016) says that any position (discourse vector) in an embedding space corresponds to a topic model; the probability of producing a given word is higher when its word vector is more similar to the discourse vector. Since each discourse atom is a position in the embedding space, we simply look at the closest words to each atom (using the cosine similarity between atom vectors and word vectors) to characterize the atom.

### Phase One Regression Model

In phase one, we built a regression model based on theory regarding social class differences in family life and expectations that family talk might correlate with family closeness. We included the demographic and individual characteristic measures and socioeconomic status measures also considered in phase two. We also included a measure of self-rated health (1 = fair or poor, 0 = good or better) and measures of family support and closeness. Family support variables included two items that asked respondents how much they can open up to their family and friends and how often do family members and friends let them down, with responses ranging from not at all (1) to a lot (4), and an item that asked “when I run into financial difficulties, I can rely on others in my family or community to support me.” Responses ranged from strongly disagree (1) to strongly agree (5). Finally, we include a rating of family closeness using a Venn diagram as a visual aid. Respondents were asked to indicate the closeness they felt to their family relative to how overlapping the circles were, ranging from no overlap (1) to almost completely overlapping (5).

**Table A.1:** List of Topics Related to Broad Topic Categories

Topic	Narrative Subtopic labels

Family	holidays, kinship, younger generation, formal transitions, family history, abuse and conflict, tracing lineage, children, transition moments, older generation
Health	well-being; injuries; psychological stress; illness and treatment; alternative medicine; psychological distress; systemic illnesses; inflammation; specialists; tumors and conditions; family health; fatigue and cold symptoms
Work	engineering and aeronautics; professional jobs; military; remuneration; facilities and related work; family members' jobs; occupations, trades; pink collar and low/middle management; industrial; transportation and machinery; therapy and rehabilitation; office work in industry; tracking processes and tools; schedules; blue collar work tasks
Religion	scriptures and learning; morality; Christianity, belief and prayer; denominations and beliefs
Criminal justice	court procedures; violence; political conflict; discrimination; police and enforcement; enforcement and intimate partner violence; emergencies and incidents; legal processes

*Source:* Authors' tabulation.
